# Taxonomy-based content analysis of sedentary behavior questionnaires: A systematic review

**DOI:** 10.1371/journal.pone.0193812

**Published:** 2018-03-06

**Authors:** Fabien Rivière, Salomé Aubert, Abdou Yacoubou Omorou, Barbara E. Ainsworth, Anne Vuillemin

**Affiliations:** 1 EA 4360 APEMAC, University of Lorraine, Paris Descartes University, Nancy, France; 2 Healthy Active Living and Obesity Research, Children’s Hospital of Eastern Ontario Research Institute, Ottawa, Canada; 3 INSERM, CIC-1433 Clinical Epidemiology, CHRU Nancy, France; 4 College of Health Solutions, Arizona State University, Phoenix, AZ, United States of America; 5 Université Côte d’Azur, LAMHESS, Nice, France; Vanderbilt University, UNITED STATES

## Abstract

**Background:**

Health effects of sedentary behaviors (SB) may vary depending on their characteristics such as type, purpose, duration, and intensity of the behavior. While a growing number of questionnaires assess sedentary behaviors, it is unclear which characteristics of SB are measured. The aim of this review was to examine the content of self-report SB questionnaires.

**Methods:**

Three databases were searched for sedentary behavior questionnaires published before January 1^st^, 2016. Based on the inclusion criteria, 82 articles out of 1369 were retrieved for a total of 60 questionnaires. For each questionnaire, the sedentary behavior characteristics identified were reported and analyzed.

**Results:**

Most of the questionnaires assessed the time (n = 60), posture (n = 54), purpose (n = 46) and the types (n = 45) of SB performed. Fewer questionnaires assessed the environment (n = 20) social context (n = 11), status (n = 2), and associated behaviors (n = 2) related to sedentary behaviors. All the questionnaires except two assessed time spent in SB with 17 assessing frequency and 6 assessing breaks in SB. The most frequent characteristics identified in the questionnaires were the categories of sitting (90%), a day (95%), watching television (65%) and using a computer (55%). Many characteristics of SB were not measured.

**Conclusions:**

By knowing the breadth of SB included in questionnaires, this review provides support to shape the design of new questionnaires designed to reduce the gaps in measuring sedentary behaviors.

## Introduction

Sedentary behaviors (SB) are defined as “as any waking behavior characterized by an energy expenditure ≤1.5 METs while in a sitting or reclining posture” [1]. Health effects of sedentary time have been studied over the past decade with most studies showing negative associations between sedentary time and health outcomes in both adults and youth [2–4]. Much of the evidence for these results has been provided by self-report [2] with the majority of the studies measuring television (TV) viewing or total sitting time derived from a single question [4,5]. However, measuring only total sedentary time may not provide enough information to understand the health effects of SB. For example, an individual can engage in different types of SB including TV viewing, using a computer, reading, writing, and eating which have several purposes including work, transportation, and leisure time. The types and purposes of SB will differ for each person studied. Some studies have shown that the associations between SB and health-related outcomes vary by the characteristics of the SB measured and in the manner in which sedentary time is accumulated [6–8]. A systematic review of the effects of SB on health outcomes showed that TV viewing had a different impact than reading on cognitive development in early childhood [9]. The investigators showed detrimental associations between the total duration and frequency of watching TV and videos and using computers and/or overall screen time with cognitive development, whereas beneficial associations were found between the total duration and frequency of reading or being read to and cognitive development. However, the associations were complex as positive associations were shown for some TV content (educational channel viewing) while negative associations were observed for other content (cartoons). These findings are supported by another systematic review examining the relationships between SB and health indicators in children and youth [10] that showed negative associations between screen-related behaviors with body composition and cardiometabolic status (TV viewing), behavioral conduct and pro-social behavior (TV viewing and video game use), physical fitness (screen time), and self-esteem (screen time and computer use). Conversely, increased duration of reading and doing homework were associated with higher academic achievement. Such relationships imply the association between SB and health outcomes is complex and that multiple characteristics of SB should be taken into consideration in research studies. Therefore, measuring the characteristics of SB is important as it may allow researchers to understand factors mediating the relationships between sedentary time and various health-outcomes, reveal insights into an individual’s behavior, relationships between various determinants and correlates of health outcomes, and implement efficient interventions to reduce SB.

As SB are complex behaviors, their assessment is a challenge. Methods used to measure SB include subjective instruments, including questionnaires, logs, and ecological momentary assessment (EMA). Objective instruments include motion- and posture sensors. Subjective instruments are used to collect qualitative information about SB including the types and purposes of SB. Because of the ease of use and low burden, questionnaires often are used to recall detailed information about SB. To advance knowledge of the effects of SB on health outcomes, it is important that multiple characteristics of SB can be assessed by questionnaires.

**Fig 1:**
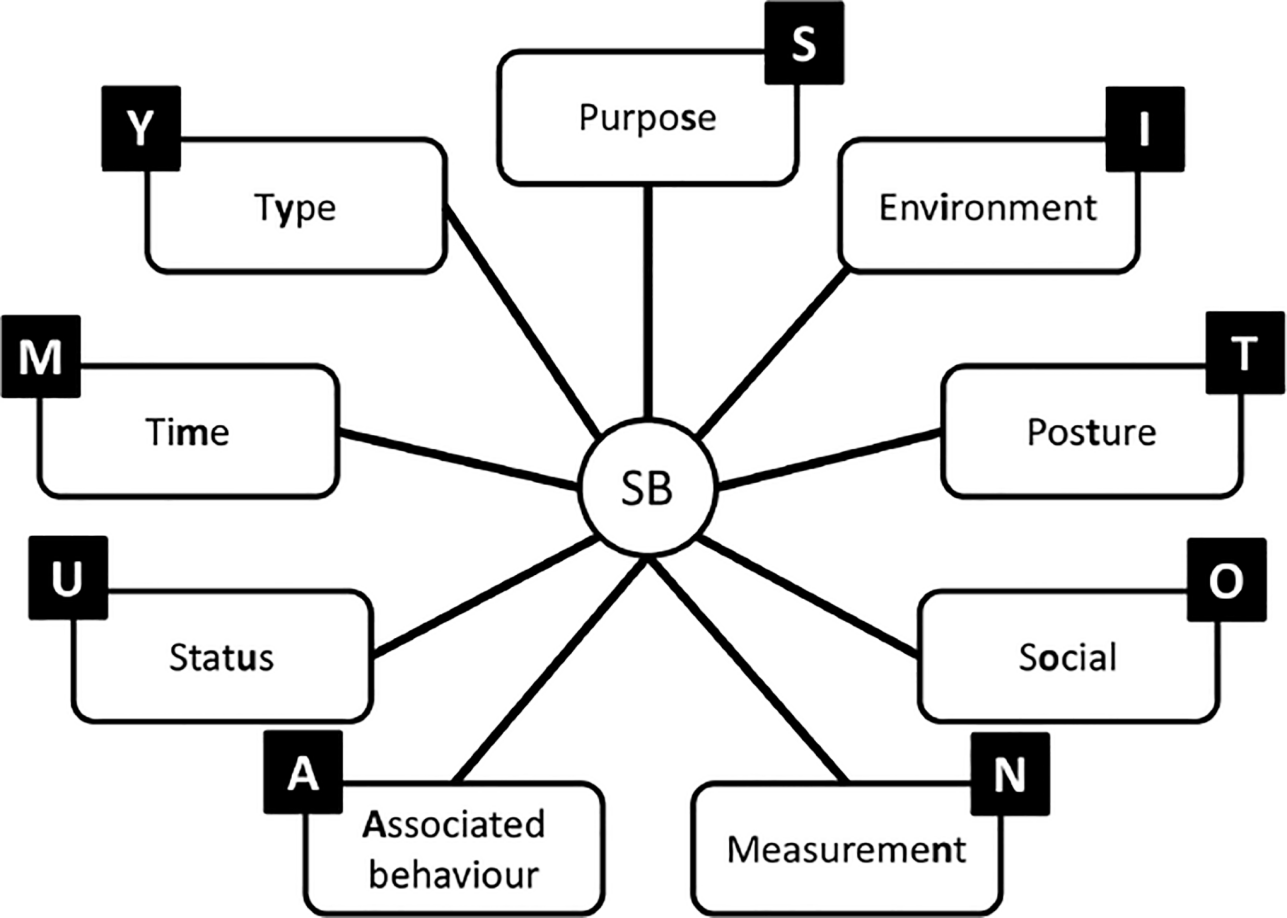
Taxonomy level one.

**Fig 2:**
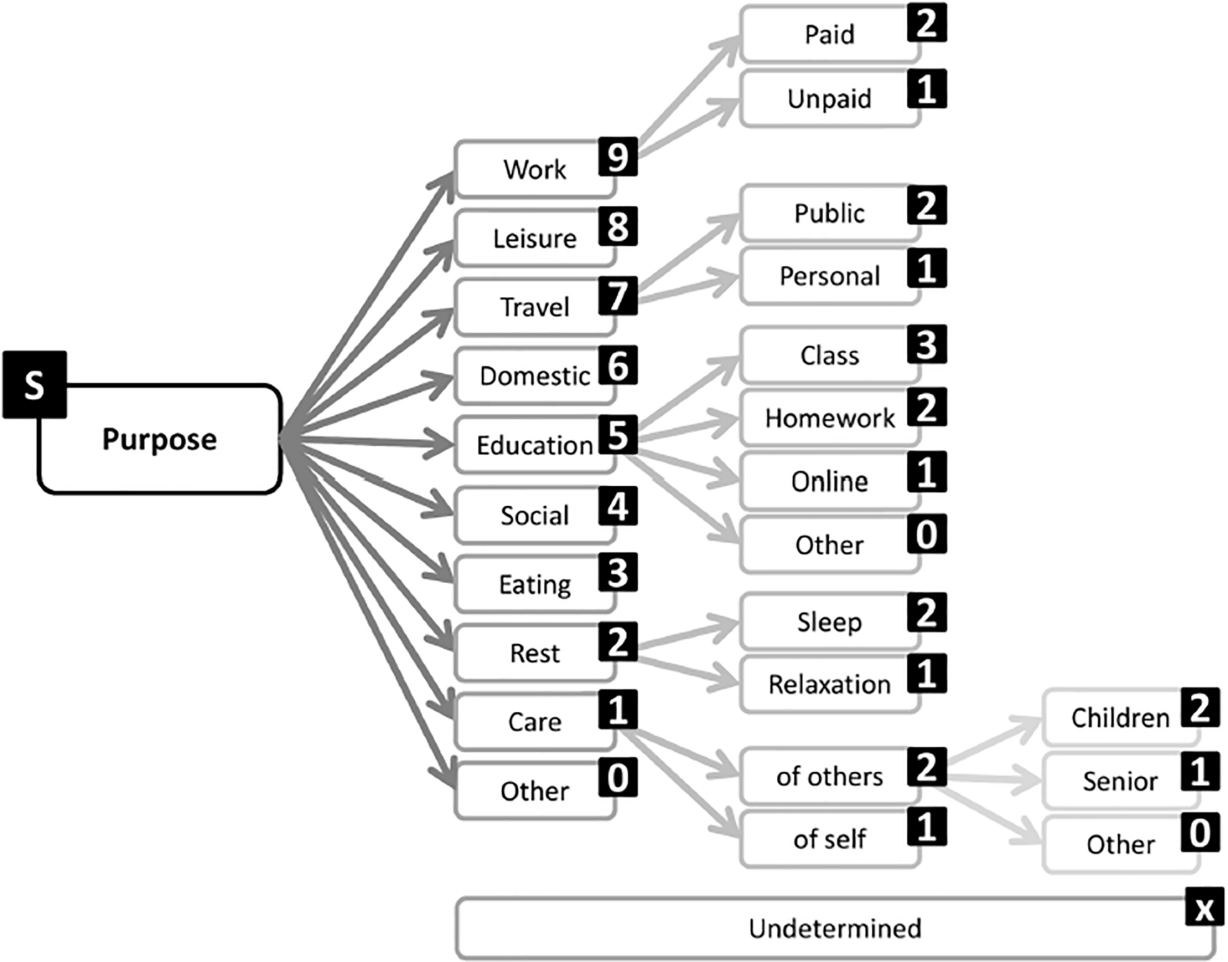
Taxonomy level one purpose and sublevels.

To better characterize SB, a taxonomy of SB was developed by Chastin and colleagues in 2013 [11]. The taxonomy of SB was the result of the first round of an open science project referred to as SIT, a term used to represent the Consensus Taxonomy of Sedentary Behaviors. This formal consensus process involved international experts who offered a comprehensive frame of reference for SB developed through a Delphi method to identify components of SB. The resulting taxonomy includes nine complementary categories (referred to as facets) and sub-categories to describe SB: posture (sitting or lying), the purpose of the behavior (such as work or for transportation), the time of day or year when one engages in SB, the types of behaviors engaged in while sedentary (no screen or screen time), the environment (community, physical, and location) and social context (alone or with others) where SB occurred, the associated behaviors (such as eating and smoking), one’s status (relating to function and psychology), and the instruments measuring the behavior (subjective, objective, and metrics) (see Figs 1 and 2 for an example of the taxonomy of SB). Questionnaires assessing SB vary considerably in length and item content. While the measurement properties of SB questionnaires have been assessed in several reviews [12,13], examination of the content of SB questionnaires in a detailed and standardized manner is warranted. Therefore, the aim of this study was to use the Taxonomy of SB to systematically appraise and compare the content of SB questionnaires. We provide information regarding the facets and categories of SB measured in published questionnaires. This information has the potential to support the development of new questionnaires that measure SB characteristics not measured currently and to reduce the gaps in measuring SB.

The objectives of this study were (1) to examine the content of questionnaires measuring SB and identify the indicators used to synthetize the information recorded, and (2) to compare the content of the questionnaires based on a well-defined and standardized classification of SB.

## Methods

This systematic review aimed to identify all studies published before January 1, 2016 that report the development and/or the psychometric properties of self-report questionnaires to assess SB. The PRISMA Statement was used to guide the report of this work [14]. The PRISMA checklist is available in the supporting information (see S1 Table).

### Literature search

The following electronic bibliographic databases were searched: Medline (PubMed), PsycINFO/ARTICLE (EBSCOhost) and SportDiscus (EBSCOhost). The full search strategies in (A) PubMed and (B) PsycINFO/ARTICLE and SportDiscus were as follows:

(A) (sedentar*[TIAB] OR Sedentary Lifestyles[MeSH] OR sitting[TIAB]) AND (questionnaires[MeSH] OR questionnaire*[TIAB] OR report*[TIAB]) AND (valid*[TIAB] OR reliab*[TIAB] OR Reproducibility of Results[MeSH])
(B) (TI(sedentar* OR sitting) OR AB(sedentar* OR sitting)) AND (TI(questionnaire* OR report*) OR AB(questionnaire* OR report*)) AND (TI(valid* OR reliab*) OR AB(valid* OR reliab*))

In addition, existing reviews of SB questionnaires were hand-searched to identify potential missing questionnaires [11, 12].

### Study inclusion and exclusion criteria

Studies meeting all of the inclusion criteria were included: (i) the aim of the study was the development of a measurement instrument or the evaluation of one or more of its measurement properties; (ii) the instrument under study was self-reported, either self-administered or administered by a researcher in the form of an interview. Proxy-reported questionnaires were excluded (proxy questionnaires are used to measure the characteristics of a subject by asking other people close to the subject such as the parents or caregiver); (iii) the instrument was a questionnaire. Use-of-time tools, logs and diaries were excluded; (iv) the questionnaire measured SB; (v) the study was accepted as a full text original article in a peer-reviewed journal until December 31, 2015; (vi) the article was published in English or French and the questionnaire was available in one of these languages.

### Study selection

Two reviewers independently assessed titles/abstracts (AV, FR) and selected full-text articles (FR, SA) based upon the eligibility criteria. In the case of a disagreement between the two reviewers, a third reviewer (AO) made the final decision. Full text copies were obtained for all but three articles meeting initial screening by one of the reviewer (FR). The reviewers were not blinded to the authors or journals when extracting data.

### Data extraction

#### Description of questionnaires

The general characteristics of the instruments were extracted from the papers using a standardized data-extraction form. This information included: (i) name of the questionnaire; (ii) version; (iii) construct to be measured; (iv) targeted age group; (v) number of items; (vi) mode of administration; (vii) recall period; (viii) dimensions; and (ix) indicators. Two reviewers independently extracted all the data. Disagreement were resolved through discussion and consensus.

#### Content of questionnaires

The content comparison aimed to identify the SB characteristics measured by each questionnaire for each item. To allow the comparison and analysis of the questionnaires, the decision was made to link the SB characteristics measured to the taxonomy of SB [13]. The taxonomy served as a reference framework to identify and classify the different categories of SB. The taxonomy of SB is composed of nine main facets (Fig 1). Each of the facets have sub-categories. For example, the level one facet “purpose” has three sub-categories (referred to as sublevel facets) as presented in Fig 2. The content of each questionnaire was systematically linked to the corresponding facets and sub-categories of the taxonomy of SB following standardized linking rules (see Table 1). A short-hand version of the taxonomy of SB was used to reduce the ambiguity of the results of the linking process by omitting “undetermined” and “others” categories. To allow the linking process, the taxonomy was used in a hierarchical structure. For each questionnaire, the following information was reported: (i) the number of items assessing SB characteristics; (ii) the number of SB characteristics identified; and (iii) the facets and categories of the taxonomy covered.

**Table 1:** Guidelines for linking questionnaires’ items to the taxonomy of SB.

Number	Rule
**1.**	Before starting the process of linking SB questionnaires to the taxonomy categories, good knowledge of the taxonomy should be acquired and all meaningful SB characteristics within each item of the questionnaire under consideration should be identify.
**2.**	Only SB characteristics should be linked. For example, “How many times a week did you travel from home to your main work?” does not assess any SB.
**3.**	Each meaningful SB characteristic within items is linked to the most precise taxonomy category.
	For example, item 6a of the STAR-Q “Driving a car or light truck” should be linked to the subcategory S71 *personal* from the category *travel* within the domain *purpose*.
**4.**	If a single item encompasses different SB characteristics, all SB characteristics should be linked. For example, in item 7a of the SIT-Q “How much time per day did you spend sitting for job?” the characteristic, *day*, *sitting and job* should be linked.
**5.**	If a SB characteristic within an item is explained by examples, both the SB characteristic and the examples should be linked. However, the taxonomy categories to which the examples have been linked should be put within parentheses. Examples often are introduced using “such as”, “for examples”, “e.g.” and/or appear in parentheses. For example, in item 1a of the WSQ “for transport (e.g., in car, bus, train, etc.)” *car* should be linked to the subcategory S71 *personal* from the category *travel*.
**6.**	The response options of an item are linked if they contain SB characteristics. For example, in item 3 of the PASBAQ “Which of these did you do whilst working? Answer options: sitting down or standing up; walking at work; climbing stairs or ladders*”*. If the response is *sitting down* then that response should be linked to the appropriate taxonomy category.
**7.**	If a SB characteristic in an item is more general than the corresponding taxonomy substructure category, the higher level of category should be linked.
**8.**	The recall period (the interval of time to which the item refers), time (the duration of the SB), the frequency (number of bouts of a certain duration) and the interruption (breaking up SB) are not linked to the taxonomy.

The linking process was inspired from the International Classification of Functioning, Disability and Health linking rules [15] and adapted to this purpose. The linking rules were developed first and then refined after being applied to some questionnaires. The final linking rules comprised of eight rules as listed in Table 1. The linking process was performed by two independent researchers who were trained in applying the taxonomy and the linking rules. Disagreement between the independent ratings was discussed until a consensus was reached.

## Results

### The literature search

**Fig 3:**
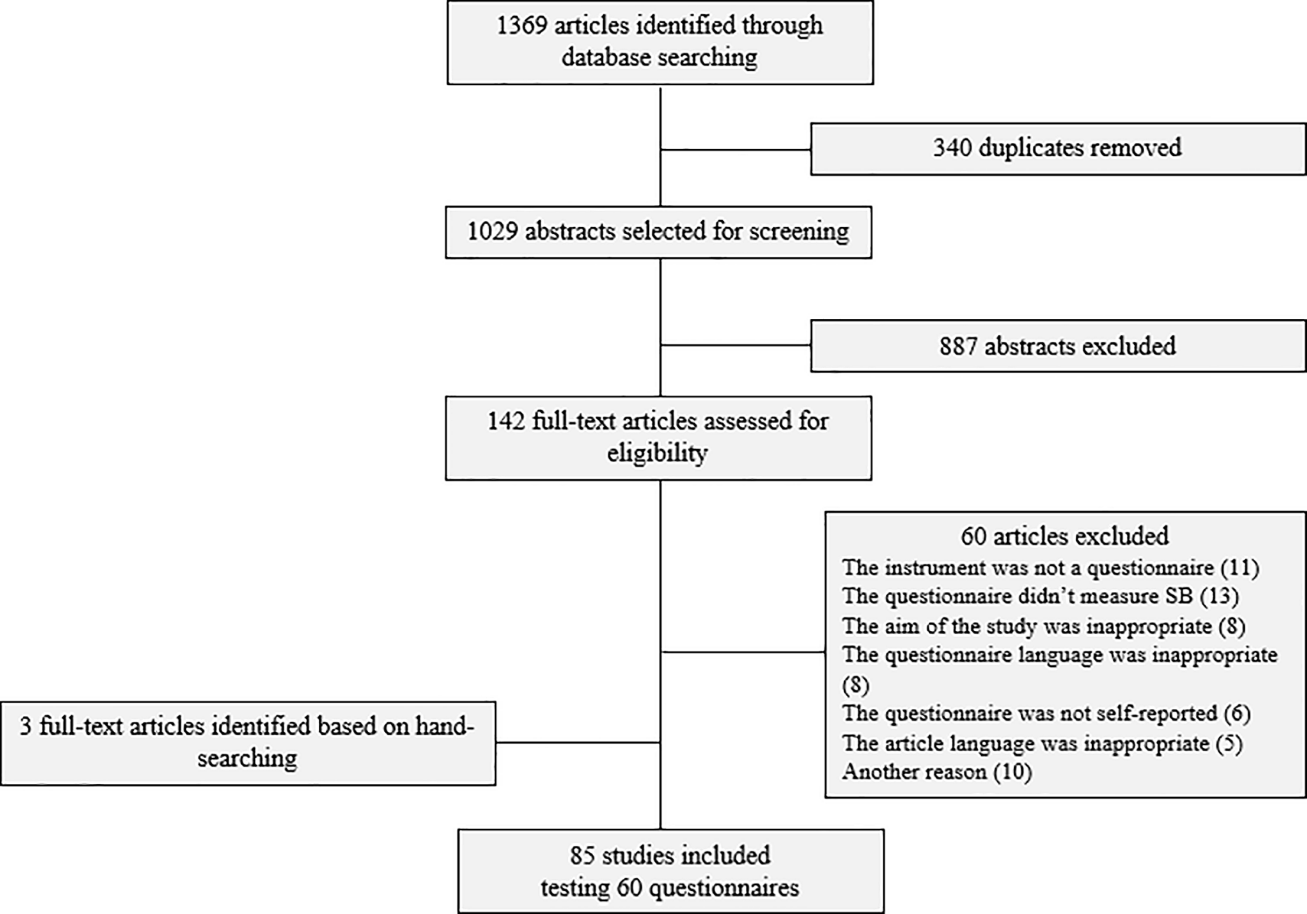
Flow chart.

The literature search produced a total of 1,369 hits: 946 in PubMed, 221 in PsycINFO/ARTICLES and 202 in SportDiscus. When selecting articles based on the inclusion criteria, 82 studies were retrieved and three additional articles were identified based on hand-searching of existing reviews for a total of 60 questionnaires. The retrieval process and the full list of questionnaire abbreviations and their corresponding definitions are presented in Fig 3 and S2 Table, respectively.

### Description of questionnaires

A description of the selected questionnaires describing SB item-characteristics is presented in Table 2. Some questionnaires included items only on SB and other questionnaires included items about SB and PA. When the questionnaires measured PA, only the SB-related content was abstracted and reviewed. From the 60 questionnaires meeting the inclusion criteria, 24 measured SB only and 36 measured both SB and PA. Questionnaires were developed and/or tested for use in the following populations: healthy adults (n = 33), adults with specific health problems (n = 11), adolescents (n = 9), seniors (n = 9), children (n = 3), women (n = 1), and students (n = 1). The majority were self-administered (n = 49) and the others were interviewer-administered (n = 25). The recall period was either a single day (n = 23) including the previous day, workday, or week-end day, a week (n = 28) including a usual week or last week, past month (n = 7), or a longer recall period (n = 6). All the questionnaires, except two which were not defined, assessed time spent in SB in hours or minutes. Seventeen questionnaires measured the frequency of SB using various metrics and six measured breaks in SB.

**Table 2:** Description of sedentary behaviors items from published questionnaires.

Questionnaire	Construct measured	Target population	Mode	Recall period	# of items	Dimensions			Indicators
						*Frequency*	*Time*	*Breaks*	
Active-Q [16]	SB, PA	Adults	SA	Past month	16	# days/week	h/day or m/day	/	MET-Time, Duration
AD3STQ [17]	SB	Adults	I	Last week	10	/	h-m/week, h-m/week-end	/	Duration
AJPAS [18]	SB, PA	Adults	SA	Average weekday	3	/	h-m/day	/	MET-Time, Duration
ASAQ [19,20]	SB	Adolescents	SA, I	Each day of a normal school week	79	# days/week	h-m/day	/	Duration
ASTSQ [21]	SB	Older adults	I	Usual weekday, usual weekend day and previous day	3	/	h/day	/	Duration
AQuAA [22,23]	SB, PA	Adolescents, Adults, Obese and overweight pregnant women	SA	Average/day during the last 7 days	11	# days/week	h-m/day	/	Duration
AWAS [24]	SB, PA	Women	I	Average day during a typical week and weekend	27	# days/week, # days/weekend	h-m/day	/	Duration
CAPANS-PA-M [25]	SB, PA	Adolescent	SA	Normal day in the past 7 days	44	/	h-m/day	/	Duration
CHAMPS [26]	SB, PA	Older adults	SA	Typical wk during the last 4 weeks	18	# times/week	h/week	/	Duration
CSIST [27]	SB	Adults	SA	Today	1	/	h-m/day	/	Duration
DSSTQ [27]	SB	Adults	SA	Usual weekday and weekend day	10	/	h-m/day	/	Duration
EAST-Q [28]	SB	Adolescents	SA	Average weekday and weekend day during the current school year/past year/summer	5	/	h/day	/	Duration
EPAQ2 [29]	SB, PA	Adults	SA	Average weekday and weekend day during the past 12 months	23	Frequency of mode of transportation (always to never)	h/week or h/day	/	Duration
GPAQ [30–32]	SB, PA	Adults	SA, I	Typical day on a typical week	1	/	h-m/day	/	Duration
HBSC [22]	SB, PA	Adolescents	I	Usual weekday and weekend day	6	/	h/day		Duration
iHSQ [33]	SB, PA	Adolescents	SA	Typical school day, average school week	14	Modes of transportation: # days/week	Minutes or hours / day	/	Duration
IPAQ-E [34]	SB, PA	Older adults	SA	Average/day during the last 7 days	1	/	h-m/day	/	Duration
IPAQ-LF [35–43]	SB, PA	Adults, Older adults, Patients with T2DM, Overweight adults	SA, I	Average/weekdays and weekend days during the last 7 days	4	# days/week in a motor vehicle	h-m/day	/	Duration
IPAQ-LF-Hausa [44]	SB, PA	Adults	SA	Average/weekdays and weekend days during the last 7 days	4	# days/week in a motor vehicle	h-m/day	/	Duration
IPAQ-LF-Fibromyalgia [45]	SB, PA	Women with fibromyalgia	SA	Average/weekdays and weekend days during the last 7 days	6	# days/week in a motor vehicle	h-m/day	/	Duration
IPAQ-LF-Inuit [46]	SB, PA	Adults	I	Average/weekdays and weekend days during the last 7days	4	# days/week in a motor vehicle	h-m/day	/	Duration
IPAQ-SF [34,41,47–57]	SB, PA	Adolescents, Adults, Older adults, Blind adults	SA, I	Average/day during the last 7 days	1	/	h-m/day	/	Duration
IPAQ-SF-Hausa [54]	SB, PA	Adults	SA	Average day during the last 7 days	1	/	h-m/day	/	Duration
LASA-SBQ [58]	SB	Older adults	SA	Average weekday and weekend day	20	/	h-m/day	/	Duration
LoPAQ [59]	SB, PA	Patients on hemodialysis	I	Average/day during the last 7 days	5	# naps/week	h/day	/	Duration
LOSTQ [60]	SB	Adults	SA	Average working and leisure day during the measuring period (7d)	8	/	h-m/day	/	Duration
MDSSTQ [61]	SB	Adults	SA	Usual weekday and weekend day	10	/	h-m/day	/	Duration
MOSPA-Q-M [62]	SB, PA	Adults	SA	Typical workday in the last 7 days	1	/	h-m/day	/	Duration
MPAQ [63]	SB, PA	Adults	I	Typical workday, weekday and week-end day	44	Frequency (daily, weekly, monthly, yearly, never)	h-m/day	/	Duration
MSTQ [64]	SB	Adults	SA	Average work day and non-work day during an usual week	14	/	h-m/day	/	Duration
OSPAQ [65]	SB, PA	Adults	SA	Typical workday in the last 7 days	3	/	%, h-m/day	/	Duration
PACI [66–68]	SB, PA	Children	I	Yesterday before and after school	4	/	h-m/day	/	Duration
Paffenbarger PAQ–Q8 [69]	SB, PA	Adults	I	Usual weekday and weekend day	4	/	h/day	/	Duration
PAQ [70]	SB, PA	Adults	I	Typical day	7	/	h-m/day	/	Duration
PASBAQ [71]	SB, PA	Adults	I	Average weekday and weekend day in the last 4 weeks	4	/	h-m/day	/	Duration
PAST [72]	SB	Women with breast cancer	I	Previous day	9	/	h-m/day	/	Duration
PAST-U [73]	SB	Adults (students)	I	Previous day	9	/	h-m/day	/	Duration
PPAQ [74]	SB, PA	Pregnant women	I	Usual day in this trimester	5	/	h/day	/	MET-Time, Duration
QAPE–S [75]	SB, PA	Children	SA	Each day of the last week	41	# days/week	/	/	Score
RADI [76]	SB, PA	Patients in primary care	SA	Typical day during the past wk, month, year	3	/	h/day	/	Score, Duration
RPAQ [77,78]	SB, PA	Adults	SA	Average/weekday and weekend day over the last 4 weeks	12	/	h-m/day	/	MET-Time, Duration
SAPAC [66]	SB, PA	Children	SA	Before and after school yesterday	4	/	h-m/day	/	Duration
SAPAC-M [79]	SB, PA	Preadolescent	I	Previous day before and after school	4	/	h-m/day	/	Duration
SAPAS [80]	SB	Adults	I	Typical day	2	/	h-m/day	Frequency (from always to never)	Duration, Frequency of breaks
SBQ [42]	SB	Overweight adults	SA	Typical weekday and weekend day	18	/	h/day	/	Duration
SBQ-Spanish [81]	SB	Patients with fibromyalgia	SA	Typical weekday and weekend day	22	/	m/day or h/day	/	Duration
SITBRQ [82]	SB	Adults	SA	Typical work day	2	/	/	# breaks/h, total time of break during the day at work	# of breaks
SIT-Q-12m [83]	SB	Adults	SA	Usual weekday and weekend day during the last 12 months	55	Frequency of eating while watching tv (always to never)	h-m/day	Frequency of breaks during work and tv viewing for leisure	# of breaks, Duration
SIT-Q-7d [84]	SB	Adults	SA	Average weekday and weekend day during the last 7 days	68	/	m/day or h/day	# breaks/day during sitting while doing occupation and watching TV	Duration
SMCPAQ [85]	SB, PA	Adults	SA	Average/day during the past year and ages 15, 30 and 50.	8	/	h/day	/	Duration
SQTV [86]	SB	Adults	SA	Usual week	1	/	h-m/day	/	Duration
STAR-Q [87,88]	SB, PA	Adults	SA	Average/day during the last 4 weeks	115	# days/past 4 weeks	h-m/day	/	Duration
STSBQ [89]	SB	Adolescents	SA	Usual weekday and usual weekend	12	/	h/day	/	Duration
SUASQ [90]	SB	Adults	I	Average work day during last week	2	/	h-m/day	# of breaks/h during sitting at work	# of breaks, Duration
SUHSQ [91]	SB	Older adults	I	Last week	7	/	h-m/week	/	Duration
VCSBQ [92]	SB	Older adults	I	Usual day during the last 7 days	21	# days/week	h-m/day	/	Duration
WAIPAQ [93]	SB, PA	Adults	I	Typical weekday, Saturday, Sunday or on average per day	5	/	h-m/day	/	Duration
WSQ [94]	SB	Adults	SA	Average working, non-working day during the last 7 days	10	/	h-m/day	/	Duration
YPAS [26,51,95,96]	SB, PA	Older adults, Adults with Schizophrenia, or schizoaffective disorders	I	Average day over the last month, last week	2	/	h/day, h-m/week	/	Score, Duration
YRBS [29]	SB, PA	Adolescents	SA	Average school day	1	/	h/day	/	Duration

SA: Self-Administered; I: Interview; #: Number; h: hours; m: minutes; %: Percentage; /: not listed

### Taxonomy-based content analysis

Overall, 567 SB characteristics were identified and linked to the taxonomy. The questionnaire content is presented in a shortened taxonomy format in Table 3 and is presented in the full taxonomy format in S3 Table. Important differences were observed in the characteristics of the SB measured. Among the 60 questionnaires reviewed, SB facets observed in descending order of frequency were: time (n = 60), posture (n = 54), purpose (n = 46), type (n = 45), environment (n = 20), social context (n = 11), status (n = 2) and associated behaviors (n = 2). The mean number of items per questionnaire was 14.2 [min–max = 1–115] and the mean number of SB characteristics measured per questionnaire was 9.5 [min–max = 2–27]. For questionnaires measuring only SB, the mean number of SB characteristics per questionnaire was 11.7 [min–max = 2–27] and questionnaires measuring both PA and SB the mean number was 8.1 [min–max = 2–23]. The most frequent SB characteristics in the questionnaires were time (in a day, 95%), posture (sitting, 90%), and type (TV, 65%; computer, 55%). Conversely, some SB characteristics were not measured including associated behaviors and most of the sub-categories for environment and status facets. Among the questionnaires reviewed, the ASAQ, SIT-Q-12m, SIT-Q-7d and STAR-Q were the most comprehensive. They included 55–115 items that measured 13–27 SB characteristics. The least comprehensive questionnaires were CSIST, IPAQ-SF and GPAQ which had only one item measuring overall sitting time. Table 3 presents a comprehensive evaluation of the taxonomy’s facets contained in each of the reviewed SB questionnaire items. The column labeled Taxonomy presents the main facets (bolded) followed by the first level of their associated sub-categories. The letters and numbers to the left of the facets reflect the system used to classify the facet and sub-categories. The facet titled measurement is omitted since all instruments were self-report questionnaires. The names of the questionnaires reviewed are abbreviated in the top row. The X and (X) symbols identify when the facets and/or sub-categories are measured by a questionnaire and when an example is given for a SB facet and/or sub-category in the questionnaire, respectively.

**Table 3:** Questionnaires ‘content linked to the taxonomy.

Taxonomy		Active-Q	AD3STQ	AJPAS	AQuAA	ASAQ	ASTSQ	AWAS	CAPANS-PA (Modified)	CHAMPS	CSIST	DSSTQ	EAST-Q
S	**Purpose**												
S9	Work	X (X)	X	X			(X)	X (X)					
S8	Leisure		(X)		X			X				X	
S7	Travel	X	X		X (X)	X (X)		X (X)	X			X	
S6	Domestic							X					
S5	Education	(X)				X		X	X	X			X
S4	Social		(X)	X	(X)	(X)	(X)			X		(X)	
S3	Eating			X								(X)	
S2	Rest	X		X		(X)							
S1	Care							X					
I	**Environment**												
Ic	Community												
Ip	Physical												
II	Location		X		(X)	X			X	X		X	X
T	Posture												
T2	Sitting	X	X	X	(X)	X	X	X	X		X	X	X
T1	Lying												
O	**Social**												
O2	With others		(X)	X	(X)	(X)	(X)			X		(X)	
O1	Alone												
A	**Associated behaviours**												
As	Smoking												
Ae	Eating		(X)										
Ad	Drinking												
An	None												
U	**Status**												
Uf	Functional												
Up	Psychology												
M	**Time**									X			
Md	Of day	X	X	X	X	X	X	X	X		X	X	X
My	Of year												X
Y	**Type**												
Yn	No screen	X	(X)	X	X (X)	X (X)	(X)	X (X)	X	X		(X)	
Ys	Screen	X	X (X)	X	X (X)	X	(X)	(X)	X	X		X (X)	X
		**EPAQ2**	**GPAQ**	**HBSC**	**iHSQ**	**IPAQ-E**	**IPAQ-LF**	**IPAQ-LF (Hausa)**	**IPAQ-LF (Fibromyalgia)**	**IPAQ-LF (Inuit)**	**IPAQ-SF**	**IPAQ-SF (Hausa)**	**LASA-SBQ**
S	**Purpose**												
S9	Work	X (X)											X
S8	Leisure				X	X	X	X	X	X	X	X	X
S7	Travel	X	X (X)	(X)	X (X)		X (X)	X (X)	X (X)	X (X)			X
S6	Domestic											X	
S5	Education			(X)	X	X	X		X	X	X		
S4	Social		X (X)	(X)		(X)	(X)	(X)	(X)	(X)	(X)	(X)	X
S3	Eating			(X)									
S2	Rest	X											X
S1	Care												
I	**Environment**												
Ic	Community												
Ip	Physical												
II	Location		X			X	X	X	X	X	X	X	X
T	**Posture**												
T2	Sitting	X	X	X	X	X (X)	X (X)	X (X)	X (X)	X (X)	X (X)	X (X)	X
T1	Lying		X			(X)	(X)	(X)	X (X)	(X)	(X)	(X)	X
O	**Social**			(X)									
O2	With others		X (X)			(X)	(X)	(X)	(X)	(X)	(X)	(X)	X (X)
O1	Alone												
A	**Associated** **behaviours**												
As	Smoking												
Ae	Eating												
Ad	Drinking												
An	None												
U	**Status**												
Uf	Functional												
Up	Psychology												
M	**Time**												
Md	Of day	X	X	X	X	X	X	X	X	X	X	X	X
My	Of year												
Y	**Type**												
Yn	No screen	(X)	(X)	(X)	X (X)	(X)	(X)	(X)	(X)	(X)	(X)	X (X)	X (X)
	Screen	X	(X)	X (X)	X (X)	(X)	(X)	(X)	(X)	(X)	(X)	(X)	X
		**LoPAQ**	**LOSTQ**	**MDSSTQ**	**MOSPA-Q (Modified)**	**MPAQ**	**MSTQ**	**OSPAQ**	**PACI**	**Paffenbarger PAQ-Q8**	**PAQ**	**PASBAQ**	**PAST**
S	**Purpose**												
S9	Work		X		X	(X)	X	X		X (X)			X
S8	Leisure		X	X									X
S7	Travel			X		X	X				X		X (X)
S6	Domestic												
S5	Education						X					(X)	
S4	Social			(X)			X						(X)
S3	Eating			(X)		X				(X)	(X)	(X)	X (X)
S2	Rest	X				X	X			X	X		
S1	Care												
I	**Environment**												
Ic	Community												
Ip	Physical												
II	Location			X				X			X		X
T	**Posture**												
T2	Sitting	X	X	X	X	X	X	X	X	X	X	X	X
T1	Lying												X
O	**Social**						X						
O2	With others			(X)									(X)
O1	Alone												
A	**Associated behaviours**												
As	Smoking												
Ae	Eating												
Ad	Drinking												
An	None												
U	**Status**												
Uf	Functional						X						
Up	Psychology												
M	**Time**												
Md	Of day	X	X	X	X	X	X	X	X	X	X	X	X
My	Of year												
Y	**Type**					X							
Yn	No screen	X		(X)		X (X)	X	(X)		(X)	(X)	(X)	X (X)
Ys	Screen	X		X (X)		X (X)	X	(X)	X	(X)	X (X)	X (X)	X (X)
		**PAST-U**	**PPAQ**	**QAPE—Semaine**	**RADI**	**RPAQ**	**SAPAC (Modified)**	**SAPAC**	**SAPAS**	**SBQ**	**SBQ (Spanish)**	**SITBRQ**	**SIT-Q-12m**
S	**Purpose**												
S9	Work	X				X				X	X		X
S8	Leisure												X
S7	Travel	X	X			X			X	X	X		X
S6	Domestic												
S5	Education	X (X)											X
S4	Social	X	X						X				
S3	Eating	X			(X)						X		X
S2	Rest										X		X
S1	Care												X
I	**Environment**												
Ic	Community												
Ip	Physical												
II	Location	X (X)	X		(X)	X			X			X	
T	**Posture**												
T2	Sitting	X	X		X	X			X	X	X	X	X
T1	Lying	X							X		X		X
O	**Social**		X										
O2	With others	X (X)							X				
O1	Alone												
A	**Associated behaviours**												
As	Smoking												
Ae	Eating												X
Ad	Drinking												
An	None												
U	**Status**												
Uf	Functional												
Up	Psychology												
M	**Time**												
Md	Of day	X	X	X	X	X	X	X	X	X	X	X	X
My	Of year												
Y	**Type**												
Yn	No screen	X (X)	X		(X)				X	X (X)	X	X	X
Ys	Screen	X	X	X (X)	(X)	X (X)	X (X)	X	X	X (X)	X		X
		**SIT-Q-7d**	**SMCPAQ**	**SQTV**	**STAR-Q**	**STSBQ**	**SUASQ**	**SUHSQ**	**VCSBQ**	**WAIPAQ**	**WSQ**	**YPAS**	**YRBS**
S	**Purpose**												
S9	Work	X	X		X		X						
S8	Leisure		X						X		X		
S7	Travel	X			X			X	X		X (X)		
S6	Domestic	X							X				
S5	Education	X			X	X			X				
S4	Social	X			(X)			X	X		(X)		
S3	Eating	X			X				X				
S2	Rest	X			X								
S1	Care	X			X								
I	**Environment**												
Ic	Community												
Ip	Physical												
II	Location				X		X		X	X	X		
T	**Posture**												
T2	Sitting	X	X	X	X		X	X	X	X	X (X)	X	X
T1	Lying	X		X	X			X					
O	**Social**	X			(X)						(X)		
O2	With others	X						X	X				
O1	Alone												
A	**Associated behaviours**												
As	Smoking												
Ae	Eating	X											
Ad	Drinking	X											
An	None												
U	**Status**												
Uf	Functional				X								
Up	Psychology												
M	**Time**			X									
Md	Of day	X			X	X	X	X	X	X	X	X	X
My	Of year		X										
Y	**Type**												
Yn	No screen	X (X)	X		X (X)		X	X	X (X)				
Ys	Screen	X (X)	X	X	X (X)	X		X	X	X	X (X)		X

X = inclusion of a facet and/or category; (X) = an example of a facet and/or category is provided in the questionnaire item

## Discussion

The aim of this review was to examine and compare the content of questionnaires measuring SB using facets and sub-categories of SB as described in Chastin et al.’s Taxonomy of SB. Overall, our review reports wide differences in the questionnaires’ content with the most comprehensive questionnaires measuring up to 27 SB characteristics while the least comprehensive questionnaires measured only one characteristic, overall sitting time. Most of the questionnaires measured sitting time spent watching TV or using a computer during a day. Since studies show that screen-related SB may be associated differently with health-related outcomes than other types of SB [10, 11], one should determine which characteristics of SB are of interest when selecting a questionnaire.

Questionnaires developed to obtain a more comprehensive measurement of SB characterizes patterns of SB during daily life by measuring more of the facets and categories in the taxonomy than less comprehensive questionnaires. The more comprehensive questionnaires allow consideration of a variety of SB when exploring relationships of SB to health outcomes. Many comprehensive questionnaires, such as the SIT-Q, the MPAQ and the STAR-Q, are structured into different sections whereby each section represents a purpose. For each purpose, the questionnaire asks about the time spent in SB or a characteristics of the SB. As an example, the SIT-Q-7d is one of the more comprehensive SB questionnaires. It consists of 68 items and measures time spent in different SB for work, transportation, domestic, education, socializing, eating and caregiving settings during a week day and a week-end day. This kind of structure is beneficial when addressing the complexity of SB. Not all facets were measured consistently. In some questionnaires, the purposes of SB performed during leisure activities were identified with follow-up questions, yet for work activities, only the overall sitting time was measured. Furthermore, some categories under the purpose facet were measured incompletely while other categories had several follow-up items. Only four questionnaires asked about caregiving and/or domestic SB, whereas 21 questionnaires asked about work SB and 19 questionnaires asked about leisure-time SB.

Other facets of SB were seldom measured by SB questionnaires including associated behaviors (queried as “what else?”), the social context (with whom?), and the status of an individual. These characteristics may be of interest to researchers as they have the potential to introduce bias in the relationship between SB and health-related outcomes. Associated behaviors, such as eating while watching TV, are associated with an increased risk of obesity [97] possibly resulting from nutritionally poor food choices influenced by TV commercials, less feeling of satiety while distracted by TV viewing, or by the replacement of physical activity by a sedentary behavior [98]. The social context seldom is considered when investigating SB and health outcomes. Both the quantity (having many social relationships vs. their relative absence) and quality (types of emotional support or conflict from others) of social relationships are associated with morbidity and mortality [99]. Thus, it can be expected that the social context during SB can influence the strength of the association between SB and health-related outcomes. Further, SB while alone may place one at a greater risk of health complications than engaging in SB with others. The facets of environment and time identify where a SB occurred and how long a SB occurred, respectively. These facets have a limited number of sub-categories. For the environment facet, the sub-category of indoor SB behaviors is measured on many SB questionnaires. The time facet includes two categories relating to SB performed during a day and a year. While time of the year (seasons) is known to affect PA, little is known about how it influences SB. Similarly, the environment has been identified as one of the main determinants of SB [100], however little information is available about the natural and built environment in which an individual engages in SB. (S3 Table).

Only two questionnaires asked about multitasking as associated behaviors. Individuals can engage in several tasks simultaneously, such as watching TV and chatting via Skype or Facebook or other behaviors. Watching TV could be associated with negative cognitive outcomes of using screen-based devices to chat with friends if it impacts poorly on well-being and self-esteem [101]. Little is known about how sedentary multitasking might pose a health risk as multitasking can have both distinct positive and negative health outcomes. It has been suggested that multitasking activities are associated with an increase in negative emotions, stress, psychological distress, and work-family conflict in women [102] and that media multitasking could be a unique risk factor for mental health problems [103]. Understanding the association between media use and mental health needs to consider the types of media people use, how they engage with the media, and the content of the media. Collectively, these concerns support the need to measure multitasking when investigating health effects of SB.

The taxonomy-based content analysis also brings to light that some of the characteristics of SB measured in many questionnaires that did not appear in the sub-categories of the taxonomy such as doing arts and crafts, talking with acquaintances, and hobbies. While it is not possible to add all SB characteristics to the taxonomy, identifying important characteristics common to many research settings could enrich the existing taxonomy. Even though SB is defined as any waking behavior characterized by an energy expenditure ≤1.5 METs while in a sitting or reclining posture [1], sleeping and taking a nap are classified as SB in the taxonomy. Similarly, a few characteristics of SB presented in the taxonomy are classified as physical activity on some questionnaires. In particular, cooking and household chores are included as a sub-category under the no-screen sub-category in the taxonomy. Based on the 2011 Adult Compendium of Physical Activities, these behaviors are assigned MET values > 1.5 and are scored as light-intensity activities in some questionnaires [104]. The sub-category making music could be classified as either a SB or a light-intensity physical activity depending on the questionnaire used. Yoga relaxation was classified as a SB by one questionnaire while its associated energy expenditure is 2.0 METs in the 2011 Adult Compendium of Physical Activities. The Taxonomy of SB and most of studies reviewed classified time spent in front of small screen devices such as a phone or music player as a SB; however, the energy cost of these devices can increase while walking or standing as seen with the mobile application Pokémon Go. Thus, asking for the posture of one’s SB would be useful to clarify the types of SB performed. These caveats aside, the boundary between SB and light-intensity physical activity is small and complex. Clarification of what constitutes a SB has reflected changes in the definition of SB over time. Given that the measurement and epidemiology of SB is a relatively new research field, efforts must be taken to harmonize and standardize the measurement of SB.

Differences in the recall frame, duration and mode of administration were observed in the SB questionnaires reviewed in this study. The most common recall frames were one week and/or one day which reflect the efficacy of short recall periods in enhancing the recall of information [105]. Longer recall frames are able to measure usual patterns of SB, however the potential for recall bias also is greater than for shorter recall periods [12]. All but two questionnaires measured time spent in SB. Depending on the questionnaire, duration was recalled either in hours and/or minutes per day as a continuous variable or in hours and/or minutes per day as a discrete variable. Among the questionnaires reviewed, 49 were validated using a self-administered paper or computer format and 25 were evaluated using an interview-administered in a face-to-face or telephone format. The mode of administration of questionnaires is important to reduce social desirability bias [106]. While this study included self-reported questionnaires only, proxy-report may be more appropriate for use in populations with limited cognitive capacities (i.e., children, intellectually-disabled persons, and older adults) due to their inability to recall the details of the questionnaire. In that case, parents, relatives or professional health care proxy reports may be appropriate to collect questionnaire information about the participant’s SB [5].

### Limitations

The use of the Taxonomy of Sedentary Behaviors to analyze the content of the questionnaires is a long and tedious process. Some SB characteristics appeared twice in the taxonomy and other characteristics had similar wording (i.e., at the workplace and for work) making the linking process difficult. The development of linking rules was an essential step to ensure that all of the questionnaires’ content was linked following the same criteria. Despite the linking rules, some content was linked differently between the two reviewers with a consensus reached after discussion. The use of the taxonomy served as a reference framework to allow a standardized comparison of the questionnaires’ content. Further, since only articles written in English and in French were reviewed and no grey literature was searched, we can’t rule out the possibility that some SB questionnaires were omitted.

## Conclusions

This study presented a standardized content analysis of 60 SB questionnaires to show the number and type of characteristics of the Taxonomy of SB measured in each questionnaire. Considerable variability was observed in the comprehensiveness of SB in the questionnaires reviewed. Questionnaires ranged from 1–115 items measuring from 2–27 SB characteristics. Facets for time, posture, purpose, and type were measured most often and facets for status and associated behaviors were measured least often. Sitting, TV viewing, and computer use were observed most often. A per day recall period was most frequent. When selecting a SB questionnaire, one should consider the measurement properties, the characteristics of SB, and the nature of information about the frequency, duration, interruptions, and recall frame. The taxonomy-based content analysis provides a useful tool to identify and compare the content of SB questionnaires as it provides a framework of SB characteristics with which to evaluate questionnaires. This review provides support for the development of questionnaires that measure SB characteristics not currently measured in existing questionnaires. These include associated behaviors performed in sedentary time, multitasking, physical and social environments, locations of SB, and the functional and psychological status of individuals performing the behaviors.

## Supporting information

S1 Table PRISMA checklist. (DOC)

S2 Table Full list of questionnaire abbreviations and their corresponding definitions. This file presents the entire list of SB questionnaires analyzed in this review, their abbreviations, and the references for each of them.
(DOCX)

S3 Table Content of sedentary behaviors questionnaires. This table presents in the column A and B the short form of the taxonomy in a hierarchical form. In the other columns are presented the SB characteristics identified within each questionnaire. The SB characteristics linked to the taxonomy are represented by an X, while the (X) represents the SB characteristics explained by examples (cf. linking rules).
(XLSX)
