# Supplementary material for: Taxonomy-based content analysis of sedentary behavior questionnaires: A systematic review
Source: PLoS One. 2018 Mar 6;13(3):e0193812. doi: 10.1371/journal.pone.0193812 (PMC5839579; doi:10.1371/journal.pone.0193812)
Supplement: S2 Table — This file presents the entire list of SB questionnaires analyzed in this review, their abbreviations, and the references for each of them. (DOCX) [file pone.0193812.s002.docx]

S2 Appendix. Full list of questionnaire abbreviations and their corresponding definitions

| Active-Q | Web-Based Physical Activity Questionnaire Active-Q |
| --- | --- |
| AD3STQ | AusDiab3 Sitting Time Questionnaire |
| AJPAS | Aadahl & Jorgensen Physical Activity Scale |
| AQuAA | Activity Questionnaire for Adults and Adolescents |
| ASAQ | Adolescent Sedentary Activities Questionnaire |
| ASTSQ | Aguilar Sitting Time Single Question |
| AWAS | Australian Women Activity Survey |
| CAPANS-PA-M | Child and Adolescent Physical Activity and Nutrition Survey, Physical Activity (Modified) |
| CHAMPS | Community Health Activities Model Program for Seniors |
| CSIST | Clemes Single Item Sitting Time |
| DSSTQ | Domain-Specific Sitting Time Questionnaire |
| EAST-Q | Project EAST Questionnaire |
| EPAQ2 | EPIC-Norfolk Physical Activity Questionnaire |
| GPAQ | Global Physical Activity Questionnaire (version 2) |
| HBSC | Health Behaviour in School aged Children |
| iHSQ | iHealth Study Questionnaire |
| IPAQ-E | International Physical Activity Questionnaire - Short Form (Modified for elderly) |
| IPAQ-LF | International Physical Activity Questionnaire - Long Form |
| IPAQ-LF-Hausa | International Physical Activity Questionnaire - Long Form (Modified in Hausa) |
| IPAQ-LF-F | International Physical Activity Questionnaire - Long form (Modified for people with fibromyalgia) |
| IPAQ-LF-Inuit | International Physical Activity Questionnaire - Long form (Modified in Inuit) |
| IPAQ-SF | International Physical Activity Questionnaire - Short form |
| IPAQ-SF-Hausa | International Physical Activity Questionnaire - Short Form (Modified in Hausa) |
| LASA-SBQ | Longitudinal Aging Study Amsterdam Sedentary Behavior Questionnaire |
| LoPAQ | Low Physical Activity Questionnaire |
| LOSTQ | Lagersted-Olsen Sitting Time Questionnaire |
| MDSSTQ | Marshall Domain-Specific Sitting Time Questionnaire |
| MPAQ | MDRF Physical Activity Questionnaire |
| MOSPA-Q-M | MONICA Optional Study on Physical Activity Questionnaire (Modified) |
| MSTQ | Multicontext Sitting Time Questionnaire |
| OSPAQ | Occupational Sitting and Physical Activity Questionnaire |
| PACI | Physical Activity Checklist Interview |
| Paffenbarger PAQ – Q8 | Paffenbarger Physical Activity Questionnaire - Question 8 |
| PAQ | Physical Activity Questionnaire |
| PASBAQ | Physical Activity and Sedentary Behavior Assessment Questionnaire |
| PAST | Past-day Adults' Sedentary Time questionnaire |
| PAST-U | Past-day Adults' Sedentary Time – University |
| PPAQ | Pregnancy Physical Activity Questionnaire |
| QAPE – S | Children Physical Activity Questionnaire - week |
| RADI | Rapid Assessment Disuse Index |
| RPAQ | Recent Physical Activity Questionnaire |
| SAPAC | Self-administered Physical Activity Checklist |
| SAPAC-M | Self-administered Physical Activity Checklist (Modified form) |
| SAPAS | South Australian Physical Activity Survey |
| SBQ | Sedentary Behavior Questionnaire |
| SBQ-Spanish | Sedentary Behavior Questionnaire (Modified in Spanish) |
| SITBRQ | Workplace Sitting Breaks Questionnaire |
| SIT-Q-12m | Last 12-month Sedentary Time Questionnaire |
| SIT-Q-7d | Last 7-day Sedentary Time Questionnaire |
| SMCPAQ | Swedish Mammography Cohort Physical Activity Questionnaire |
| SQTV | Survey Question on Television Viewing |
| STAR-Q | Sedentary Time and Activity Reporting Questionnaire |
| STSBQ | Screen Time-based Sedentary Behaviour Questionnaire |
| SUASQ | Stand Up Australia Study Questionnare |
| SUHSQ | Stand Up for your Health Sedentary Questionnaire |
| VCSBQ | Van Cauwenberg Sedentary Behaviors Questionnaire |
| WAIPAQ | Western Australian Incidental Physical Activity Questionnaire |
| WSQ | Workforce Sitting Questionnaire |
| YPAS | Yale Physical Activity Survey |
| YRBS | Youth Risk Behviorur Questionnaire 1999 |
